# The Far-Infrared Spectrum of Methoxymethanol (CH_3_–O–CH_2_OH): A Theoretical Study

**DOI:** 10.1021/acsearthspacechem.4c00053

**Published:** 2024-05-29

**Authors:** Dorsaf Missaoui, Sinda Brahem, Faouzi Najar, Ounaies Yazidi, María Luisa Senent

**Affiliations:** † Laboratoire de Spectroscopie Atomique Moléculaire et Applications, Faculté des Sciences de Tunis, Université de Tunis El Manar, Tunis 2092, Tunisia; ‡ Departamento de Química y Física Teóricas, Instituto de Estructura de la Materia, IEM-CSIC, Serrano 121, Madrid 28006, Spain; § Unidad Asociada GIFMAN, CSIC-UHU, Huelva 21071, Spain

**Keywords:** methoxymethanol, LAM, rovibrational, ISM, VOC, internal rotation

## Abstract

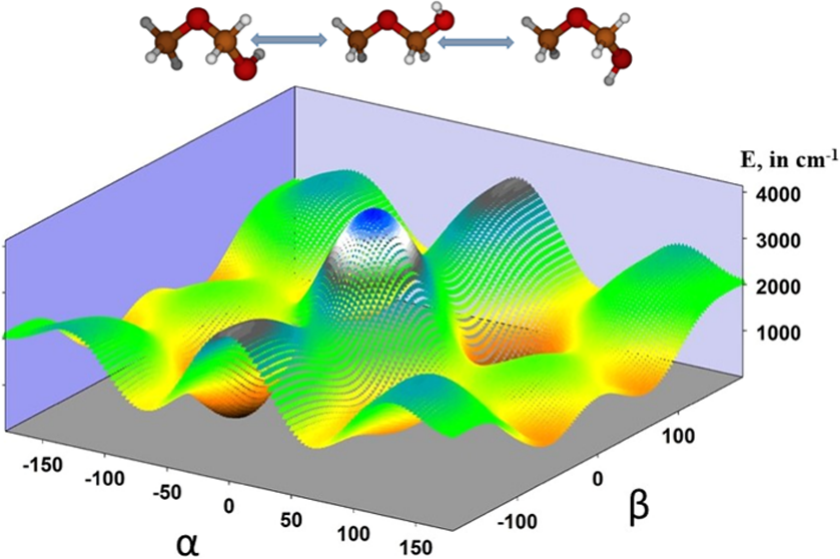

Methoxymethanol (CH_3_OCH_2_OH), an oxygenated volatile organic compound of low stability detected in the interstellar medium, represents an example of nonrigid organic molecules showing various interacting and inseparable large-amplitude motions. The species discloses a relevant coupling among torsional modes, strong enough to prevent complete assignments using effective Hamiltonians of reduced dimensionality. Theoretical models for rotational spectroscopy can improve if they treat three vibrational coordinates together. In this paper, the nonrigid properties and the far-infrared region are analyzed using highly correlated ab initio methods and a three-dimensional vibrational model. The molecule displays two *gauche*–*gauche* (CGcg and CGcg′) and one *trans*–*gauche* (Tcg) conformers, whose relative energies are very small (CGcg/CGcg′/Tcg = 0.0:641.5:792.7 cm^–1^). The minima are separated by relatively low barriers (1200–1500 cm^–1^), and the corresponding methyl torsional barriers *V*_3_ are estimated to be 595.7, 829.0, and 683.7 cm^–1^, respectively. The ground vibrational state rotational constants of the most stable geometry have been computed to be *A*_0_ = 17233.99 MHz, *B*_0_ = 5572.58 MHz, and *C*_0_ = 4815.55 MHz, at Δ*A*_0_ = −3.96 MHz, Δ*B*_0_ = 4.76 MHz, and Δ*C*_0_ = 2.51 MHz from previous experimental data. Low-energy levels and their tunneling splittings are determined variationally up to 700 cm^–1^. The *A*/*E* splitting of the ground vibrational state was computed to be 0.003 cm^–1^, as was expected given the methyl torsional barrier (∼600 cm^–1^). The fundamental levels (100), (010), and (001) are predicted at 132.133 and 132.086 cm^–1^ (methyl torsion), 186.507 and 186.467 cm^–1^ (O–CH_3_ torsion), and 371.925 and 371.950 cm^–1^ (OH torsion), respectively.

## 1 Introduction

Methoxymethanol (CH_3_OCH_2_OH, MeOCH_2_OH) is a highly reactive oxygenate organic molecule that is both an ether and an alcohol that can exist in gas phase sources. In the Earth’s atmosphere,^1^ it can be originated by oxidative processes of simple ethers by radicals. Recently, in 2017, MeOCH_2_OH was detected in the interstellar medium (ISM) toward the MM1 core in the high-mass star-forming region NGC 6334I,^2^ where it was found to be ∼34 times less abundant than methanol and significantly higher than predicted by astrochemical models.

On the basis of plausible formation pathways from hydroxymethyl radical and other observed radicals^3^ (i.e., CH_3_O + CH_2_OH), and from observed small organic molecules and radicals (i.e., CH_3_O + H_2_CO),^4^ methoxymethanol was suggested to be a detectable astrophysical species before been unambiguously observed in the ISM.^2^ The spatial distribution analysis of complex organic molecules in sources such as NGC 6334I reveals that the distribution of MeOCH_2_OH is notably similar to CH_3_OH, supporting that methanol represents a possible critical precursor of MeOCH_2_OH which can be produced in radical–radical reactions within interstellar ices.^5,6^ Since methoxymethanol has been derived in experiments where methanol is exposed to low-energy electrons, it has been proposed to be a good tracer of cosmic-ray-induced chemistry in the ISM.^7,8^ Grain-surface hydrogenation and O(1D) insertion reactions have been postulated as potential formation pathways.^2,9^

At room temperature and pressure, MeOCH_2_OH is a flammable liquid, for which the boiling point is 82.5 °C and the flash point is 39.9 °C.^10^ Given its instability, laboratory studies in the gas phase are not frequent. The synthesis of the isolated compound requires to follow sophisticated procedures.^11^ Experimentally, it is produced in ternary liquid mixtures of formaldehyde–water–methanol in the 298–383 K range of temperatures where methanol acts as a stabilizer.^12^ Furthermore, it has been observed as a primary product of continuous methanol oxidation in the near-surface gas phase over all Pd-based catalysts where it is considered a key intermediate in the production of methyl format.^13^

In addition to the astrophysical interest, methoxymethanol is involved in relevant atmospheric processes that originated from dimethyl ether (DME). The interest in the atmospheric chemistry of DME^1,14^ and its derivatives is due to their use as diesel fuel substitutes since their emissions to the atmosphere might be harmful to the environment. DME tropospheric oxidation is mainly initiated by the reaction with hydroxyl radicals leading to the methoxymethyl radical which can produce MeOCH_2_OH through a series of oxidative processes with NO and O_2_, where peroxy radicals RO_2_ such as methoxymethyl peroxy radical (CH_3_OCH_2_O_2_) act as intermediates.^1^ The reaction of DME with atomic oxygen generated by photolysis of ozone or N_2_O has been examined in low-temperature matrices which makes it interesting for the study of the chemical behavior of pollutants.^14^ The major reaction products are the two most stable conformers of MeOCH_2_OH. Reactions are analyzed using ab initio calculations.^14^

The infrared spectrum of methoxymethanol was first reported in 1991 by Johnson et al.,^15^ who used an unconventional gas chromatographic/Fourier transform infrared (GC/FT-IR) technique recording the spectra with a resolution of 1.0 cm^–1^. Later on, in 1999, the IR absorption spectrum of the main isotopologue of CH_3_OCH_2_OH and five isotopic species, CH_3_–O–CH_2_O^18^H, CH_3_–O^18^-CH_2_OH, CH_3_–O^18^-CH_2_O^18^H, CD_3_-O-CD_2_-OD, and CD_3_-O-CD_2_O^18^D, were measured in argon matrices at 10 K by Wrobel et al.^14^ They assigned the bands observed between 575 and 3700 cm^–1^, to the two more stable conformers with the help of density functional theory (DFT) calculations. For the first two conformers, the OH stretching fundamentals of the main isotopologue were observed at 3631 and 3641 cm^–1^, respectively. In the most stable conformer, the most intense absorption band was attributed to skeletal bending. The low-lying transition, observed at 576 cm^–1^, was assigned to the COC bending.

MeOCH_2_OH is a nonrigid molecule where three large-amplitude motions (LAMs) interconvert different conformers separated by low energy barriers. Recently, the rotational spectrum was measured over the frequency ranges of 150–200, 220–330, and 400–460 GHz, and assigned by Motiyenko et al.^2,11^ with the aid of MP2/aug-cc-pVTZ^16,17^ ab initio calculations which supplied three different conformers very close in energy. The analysis^11^ was essential for the astrophysical detection of the most stable conformer^2^ which shows a very small dipole moment. The methyl group internal rotation was explicitly considered in the assignment of the spectrum of the most stable structure. For the third conformer, OH torsional motion was contemplated. The assignments concerning the intermedium equilibrium structure were tentative due to challenges derived from the effects of multiple interactions among LAMs. Motiyenko et al.^11^ estimated the methyl torsional barrier to be *V*_3_ = 545.92 (39) cm^–1^.

Methoxymethanol conformers interconvert through the internal rotation of the methyl group, the OCH_3_ methoxy group, and the hydroxyl group. As it has been experimentally derived,^11^ following a behavior common to many organic molecules, interactions between torsional modes are not negligible. This problem is rarely contemplated in the construction of effective Hamiltonians for the analysis of experimental rotational spectra. In the present paper, the far-infrared spectrum of methoxymethanol is explored by solving variationally a three-dimensional Hamiltonian depending on the three internal rotations. Low-lying vibrational levels and their splittings are determined. All of the minima and the three vibrational modes responsible for their interconversion modes are treated together. The parameters of the Hamiltonian are derived using highly correlated ab initio calculations, searching for very accurate properties that can be useful for the interpretation of further experimental studies.

The variational model has been employed for previous studies of other organic nonrigid species such as ethylene glycol,^18,19^ isopropyl alcohol,^20^ peroxyacetic acid,^21^ and hydroxyacetone, CH_3_COCH_2_OH.^22^ This last ketone shares properties with the MeOCH_2_OH ether, in the same way as acetone and DME, which show two interacting methyl groups.^23,24^ In this paper, we compare the behavior of these pairs of molecules. The energy levels allow us to compute the vibrational partition functions required by the radiative transfer models used for the interpretation of astrophysical observations.^25^

## 2 Results and Discussion

### 2.1 Electronic Structure Calculations

The full optimization of the methoxymethanol equilibrium structures and the computation of the energies used to build the three-dimensional energy surface (3D-PES) were achieved using the explicitly correlated coupled cluster theory with single and double substitutions augmented by a perturbative treatment of triple excitations, (CCSD(T)-F12b)^26,27^ as it is implemented in MOLPRO version 2022.^28^ Default options were employed. The theoretical procedure was applied in connection to the cc-pCVTZ-F12 basis set^29^ (denoted in this paper by CVTZ-F12) optimized for accurately describing core–core and core–valence correlation effects. All of the electrons were correlated in the post-SCF process.

Anharmonic frequencies and anharmonic contribution to the rotational constants were obtained using the vibrational second-order perturbation theory (VPT2)^30^ implemented in GAUSSIAN 16 version C.01.^31^ The force field was obtained with second-order Möller–Plesset theory (MP2)^16^ in connection with the aug-cc-pVTZ basis set (denoted in this paper as AVTZ).^17^

The large-amplitude motions and the far-infrared region were explored using a variational procedure of reduced dimensionality, which takes the minimum interconversion into consideration. The procedure and the corresponding results are described in the last section of this paper. Details can be found in previous papers.^19,32,33^

### 2.2 Structure of Methoxymethanol

**Figure 1:**
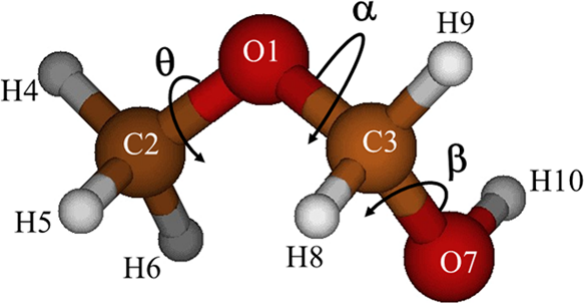
Most stable conformer of MeOCH_2_OH.

MeOCH_2_OH is a very flexible molecule where three internal rotations, the methyl group torsion (θ), the torsion of the methoxy group OCH_3_ (α), and the hydroxyl group torsion (β) interconvert all of the equilibrium geometries. Figure 1 can help to understand the torsional coordinates and the labeling of the atoms.

The three torsional coordinates θ, α, and β are defined in this paper as linear combinations of curvilinear internal coordinates
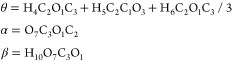
1

Three dihedral angles are combined to produce the θ methyl torsional coordinate because the CH_3_ group loses the *C*_3*V*_ symmetry when geometry is optimized. This behavior arises from the molecular structure because H_4_ almost lies in the C_2_O_1_C_3_ plane, whereas H_5_ and H_6_ are “out-of plane” atoms. In addition, the interaction with the CH_2_OH group is different for the 3H methyl atoms. However, from a dynamic point of view, the three H atoms are undiscernible to obtain accurate *V*_3_ barriers and methyl torsional energies implied to define a symmetry coordinate.

The search of equilibrium structures at the CCSD(T)-F12/CVTZ-F12b level of theory leads to three conformers denoted by CGcg, CGcg′ and Tcg (structures I, II, and III, in ref (11)), whose properties are summarized in Table 1. The resulting structures are coherent with those previously computed with less correlated ab initio methods.^11,14^ The capital symbols CG (*cis*–*gauche*) and T (*trans*–*gauche* = ∼*trans*) designate the relative orientations of the CH_2_OH and CH_3_ groups, whereas cg and tg refer to the *cis*–*gauche* and *trans*–*gauche* relative orientations of H10 and O1. The three conformers build a ground electronic state potential energy surface of a total of 18 wells because the gauche structures correspond to double minima (θ, α, β) and (−θ, −α, −β) and the methyl group to a triple minimum. The optimized geometries are provided in the Supporting Information (see Table S1).

**Table 1:** CCSD(T)-F12/CVTZ-F12 Relative Energies (*E*, *E*^AZPVE^, in cm^–1^), Internal Rotation Barriers (*V*_3_, *V*^OCH_3_^, and *V*^OH^, in cm^–1^), Equilibrium Rotational Constants (in MHz), and Structural Parameters (Distances in Å; Angles in Degrees); MP2/AVTZ Dipole Moment (in *D*)

	CGcg	CGcg′	Tcg		CGcg	CGcg′	Tcg
*E*	0.0^a^	695.6	905.4	*A*_e_	17,322.67	17,222.28	32,782.94
*E*^AZPVE^	0.0^b^	641.5	792.7	*B*_e_	5642.84	5698.85	4389.33
θ	178.0	172.1	179.0	*C*_e_	4875.27	4823.58	4109.01
α	67.7	68.5	181.4	μ_a_	–0.2193	–0.7986	1.5608
β	64.6	–86.2	59.0	μ_b_	–0.0955	1.2717	1.0643
*V*_3_	595.7	829.0	807.5	μ_c_	0.1209	–2.1521	–1.3328
*V*^OH^ (CGcg → CGcg′)	1237			μ	0.2681	2.6242	2.3120
*V*^OH^ (Tcg → Tcg′)	1260						
*V*^OCH_3_^ (CGcg → Tcg)	1363						
C_2_O_1_	1.4166	1.4104	1.4096	O_7_C_3_O_1_	113.1	113.7	109.2
C_3_O_1_	1.3932	1.3938	1.4041	H_8_C_3_O_1_	110.9	106.0	108.9
H_4_C_2_	1.0937	1.0957	1.0853	H_9_C_3_O_1_	110.9	109.6	110.7
H_5_C_2_	1.0855	1.0855	1.0942	H_10_O_7_C_3_	107.7	109.2	107.8
H_6_C_2_	1.0898	1.0922	1.0947	H_4_C_2_O_1_C_3_	59.1	53.1	179.0
O_7_C_3_	1.4032	1.4036	1.3845	H_5_C_2_O_1_H_4_	118.9	119.0	119.2
H_8_C_3_	1.0894	1.0849	1.0994	H_6_C_2_O_1_H_4_	–121.3	–122.0	–119.4
H_9_C_3_	1.0925	1.0972	1.0932	O_7_C_3_O_1_C_2_	67.7	68.5	101.4
H_10_O_7_	0.9599	0.9579	0.9598	H_8_C_3_O_1_O_7_	122.3	117.0	122.5
C_3_C_2_O_1_	112.6	112.8	111.2	H_9_C_3_O_1_O_7_	–118.0	–123.7	–117.2
H_4_C_2_O_1_	110.5	110.9	107.3	H_10_O_7_C_3_O_1_	64.6	–86.2	59.0
H_5_C_2_O_1_	106.9	107.3	111.2				
H_6_C_2_O_1_	111.3	111.5	111.3				

a *E* = −230.248984 au.

b *E* = −230.163823 au; the anharmonic AZPVE has been computed using VPT2.

**Figure 2:**
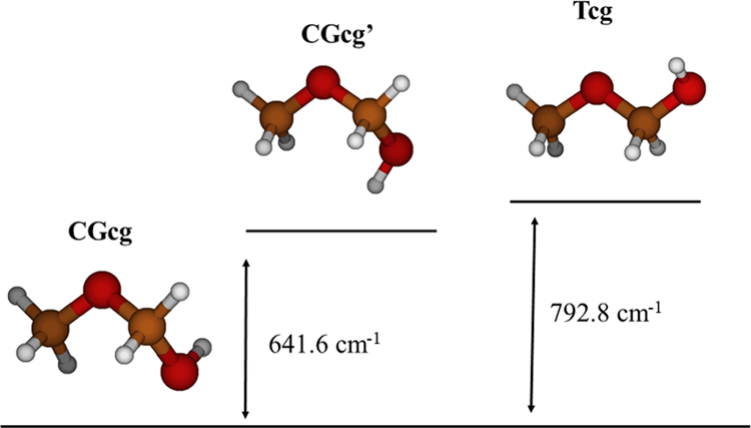
Conformers of MeOCH_2_OH. Vibrationally corrected relative energies.

Figure 2 shows the three equilibrium structures computed with the CCSD(T)-F12/CVTZ-F12b theory. Slight nonbonding interactions between hydrogen atoms determine relative stabilities. CGcg represents the favorite geometry although its stability is not prominent. Small relative energies referring to the most stable geometry were found to be 695.6 cm^–1^ (CGcg′) and 905.4 cm^–1^ (Tcg). The energies are even smaller if the anharmonic zero-point vibrational energy (AZPVE) is considered (641.5 cm^–1^ (CGcg′) and 792.7 cm^–1^ (Tcg)). In the three asymmetric conformers, θ ∼ 180°.

As stated in a previous work,^22^ the alike ketone hydroxylamine (MeCOCH_2_OH) displays four different conformers designated by Cc, Tt, Tc, and Ct. Cc is the most stable one. With the exception of Tt, which shows a symmetry plane, they are asymmetric structures. Cc, Tc, and Ct can be correlated with the CGcg, Tcg, and CGcg′ conformers of MeOCH_2_OH. The ketone is less flexible, and its conformer relative energies are larger than in methoxymethanol. They were computed to be 1206.6 cm^–1^ (Tt), 1405.1 cm^–1^ (Tc), and 2074.2 cm^–1^ (Ct), respectively.^22^ By comparing the behaviors of hydroxylamine and methoxymethanol, we obtain the same similarities and differences as when DME (ether) and acetone (ketone)^23,24^ are compared. In methoxymethanol, the stability of the secondary minima and the interconversion through low barriers are noticeable.

**Figure 3:**
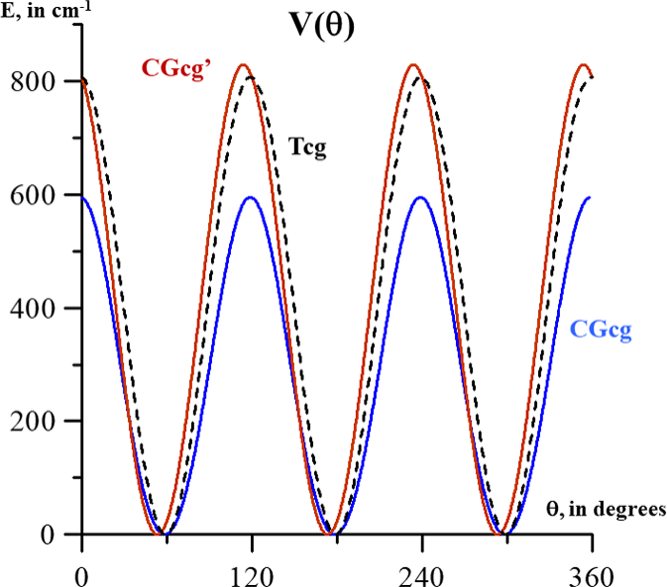
*V*_3_ methyl torsional barriers computed using CCSD(T)-F12/CVTZ-F12.

Energy profiles depending on θ were computed at the CCSD(T)-F12 level of theory by fixing the angles α and β to their respective values in the three conformers. The profiles are shown and compared in Figure 3. The torsional barriers *V*_3_ were estimated to be 595.7, 829.0, and 807.5 cm^–1^, in CGcg, CGcg′, and Tcg, respectively. In CGcg′, showing the high barrier *V*_3_, the OH hydrogen atom faces the methyl hydrogen atoms.

The *V*_3_ barriers of MeOCH_2_OH conformers (595.7:829:807.5 cm^–1^) can be compared with those of hydroxyacetone (72:472:340:57 cm^–1^).^22^ The rate *V*_3_^CH_3_OCH_2_OH^/*V*_3_^CH_3_COCH_2_OH^ is of the order of magnitude of *V*_3_^DME^/*V*_3_^Acetone^ = 990:245 cm^–1^.^23,24^ Methyl torsional barriers are much higher in ethers than in ketones. For comparison, we provide barriers of other molecules containing similar functional groups such as isopropyl alcohol,^20^ peroxyacetic acid,^21^ methanol,^34^ and methyl acetate^35^ computed to be ∼1170, ∼89, ∼378, and ∼413 cm^–1^, respectively.

**Figure 4:**
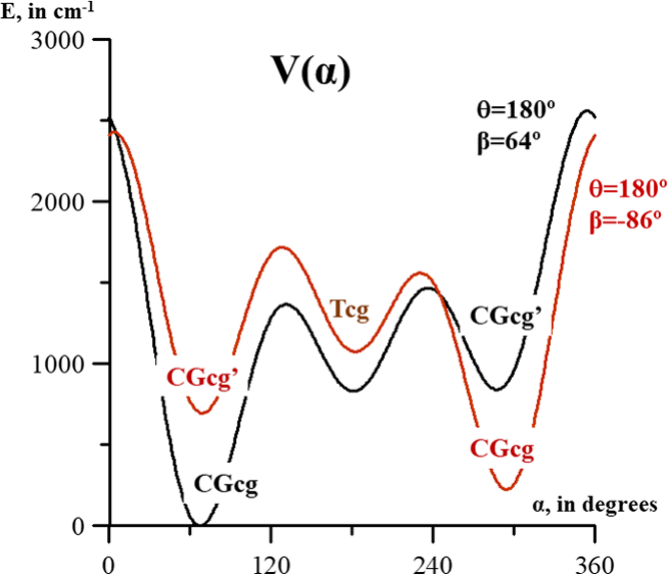
One-dimensional cuts of the potential energy surfaces depending on the coordinate α (OCH_3_ torsion). In black, the curve computed by fixing θ at 180.0° and β at 64°, and in red, θ at 180.0° and β at −86°.

Figure 4 depicts energy profiles depending on the α coordinate (O–CH_3_ torsion) computed by fixing the θ and β angles to their values in CGcg (black curve) and CGcg′ (red curve). They represent pathways for minimum interconversion that are restricted by very low barriers.

Finally, profiles 5A and 5B represent the energy variation with respect to the OH internal coordinate, β, obtained by fixing θ and α to their values in the low-energy conformer involved in the pathways. The A curve follows the CGcg → CGcg′ interconversion, which is restricted by a barrier of *V*^OH^ = 1237 cm^–1^. The B curve, corresponding to the interconversion of Tcg^+^ → Tcg^–^, is restricted by a barrier of *V*^OH^ = 1260 cm^–1^.

It is remarkable that barriers restricting the interconversion processes are really low, as a consequence of the flexibility. For example, CGcg → CGcg′ is restricted by a barrier of ∼1237 cm^–1^, but the inverse process CGcg′ → CGcg requires to reach a barrier of only ∼641 cm^–1^, which is lower than *V*_3_(CGcg′) = 829 cm^–1^. Although the CGcg → Tcg interconversion is restricted by a barrier of ∼1363 cm^–1^, the inverse process Tcg → CGcg is hindered by a barrier of only ∼570 cm^–1^, which is lower than *V*_3_(Tcg) = 807 cm^–1^. These energies justify why Motiyenko et al.^11^ did not assign the rotational spectrum of the second conformer (CGcg′) after concluding that several interacting LAMs significantly hinder assignments.

### 2.3 Full-Dimensional Anharmonic analysis

For all 24 vibrational modes, the VPT2 anharmonic fundamental frequencies are shown in Table 2. The computed transitions corresponding to the CGcg and CGcg′ forms are compared with the observed bands assigned by Wrobel et al.^14^ who measured the IR spectrum in the argon matrix. Unfortunately, to the best of our knowledge, there are no available data measured in the gas phase. The available experimental data can be considered to be consistent with our computed wavenumbers, taking into account that theoretical predictions assume the molecule to be isolated. Calculated transitions for which the expected displacements by Fermi and Darling–Dennison resonances larger than 3 cm^–1^ are highlighted in bold. Available experimental relative intensities are compared to computed relative intensities.

**Table 2:** MP2 Anharmonic Fundamentals (*ν*, in cm^–1^)^a^ and Relative Intensities (*I*)^b^ of the CH_3_OCH_2_OH Conformers

		CGcg				CGcg′				Tcg
	assig^c^	Calc. (this work)		Exp^d^		Calc (this work)		Exp^14^^d^		Calc. (this work)
		*ν*	*I*	*ν*	*I*	*ν*	*I*	*ν*	*I*	*v*
1	OH-st	3648	26	3631	18	3675	14	3641	11	3650
2	CH_3_-st	3055	12			3078	8			3053
3	CH_2_–st	**2995**	23			**3021**	10	3006	14	2971
4	CH_3_-st	3017	6	2964	13	**2962**	10	2946	12	2946
5	CH_2_–st	2927	23	2929	15	**2828**	6			**2857**
6	CH_3_-st	**2848**	9	2878	13	**2889**	25	2824	14	**2795**
7	CH_2_–b	1501	0			1493	2			1518
8	CH_3_-b	**1481**	4	1470	6	1484	1	1470	10	1486
9	CH_3_-b	**1463**	3	1452	7	1467	2	1452	6	1469
10	CH_3_-b	1450	2	1444	4	1446	1	1444	6	1452
11	O–CH_2_	1408	15	1406	3	1407	7	1414	5	1419
12	OH-b	1351	6	1355	10	1352	6	1356	15	1352
13	CH_3_-b	1277	5	1286	9	**1277**	5	1285	12	1236
14	O–CH_3_-st	1186	24	1187	34	1192	27	1186	43	1204
15	O–CH_3_-st	1159	2	1150	2	1158	2	1143	2	1167
16	O–CH_2_–st	1127	63	1125	100	1112	41	1119	55	1119
17	O–CH_3_-st	1039	75	1044		1042	5	1064	17	1103
18	O–CH_2_–st	1019	100	1020	94	**1015**	100	1019	100	1064
19	C–O-st	928	21	936	20	931	15	940	37	959
20	OCO-b	572	11	576	14	569	2	571	6	519
21	COC-b	**461**	13			**415**	1			**443**
22	OH-tor	**260**	57			**224**	27			**148**
23	OCH_3_–tor	194	5			135	3			109
24	CH_3_-tor	135	2			187	8			**216**

a Emphasized in black, the frequencies remarkably displaced by Fermi resonances.

b Relative intensity based on the strongest absorption.

c st = stretching; b = bending; tor = torsion.

d Measured in the argon matrix.^14^

The frequencies corresponding to the three internal rotations have been computed to be 135 cm^–1^ (υ_24_, CH_3_ torsion), 194 cm^–1^ (υ_23_, OCH_3_ torsion), and 260 cm^–1^ (υ_22_, OH torsion) in the most stable conformer. Resonances displace the transitions υ_22_, υ_21_ (COC bending), and 2υ_23_ in the CG conformers. In Tcg, resonances displace υ_24_, υ_22_, υ_21_ (COC bending), and 2υ_24_ υ_23_.

### 2.4 Rovibrational Parameters

The ground vibrational state rotational constants *A*_0_, *B*_0_, and *C*_0_ of the three conformers reported in Table 3 were computed using the following equation
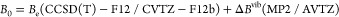
2

**Table 3:** Ground Vibrational State Rotational Constants (Computed with Equation 2) and MP2/AVTZ Centrifugal Distortion Constants

	CGcg		CGcg′	Tcg		
	Calc.^a^	Exp.^11^	Calc.^a^		Calc.^b^	Exp.^11^
*A*_0_	17233.99	17237.9490(12)	17185.35	*A*_0_	32385.67	32328.14(10)
*B*_0_	5572.58	5567.81516(29)	5608.82	*B*_0_	4350.21	4350.32898(47)
*C*_0_	4815.55	4813.04186(33)	4761.97	*C*_0_	4071.14	4070.97615(46)
Quartic Centrifugal Distortion Constants (in kHz)						
Δ*_J_*	6.0771	6.164192(49)	7.1450	*D_J_*	1.0697	1.073687(51)
Δ*_K_*	116.9145	129.292(11)	136.9866	*D_K_*	120.5840	121.2
Δ*_JK_*	–32.6575	–34.8335(13)	–42.0459	*D_JK_*	–3.4328	–3.3717(26)
δ*_J_*	1.7789	1.827024(91)	2.24547	*d*_1_	–0.1026	–0.10047(11)
δ*_K_*	15.5737	16.7256(42)	16.4989	*d*_2_	–0.0012	–0.001644(55)
Sextic Centrifugal Distortion Constants (in Hz)						
ϕ*_K_*	5.1141	6.582(34)	7.2261	ϕ*_K_*	–0.8828	
ϕ*_JK_*	0.1559	0.18786(39)	0.2365	ϕ*_JK_*	–0.0019	
ϕ*_KJ_*	–1.9621	–2.2964(47)	–2.5576	ϕ*_KJ_*	–0.4893	
ϕ*_j_*	0.0025	0.0397(24)	–0.0287	ϕ*_j_*	0.0000	
ϕ*_jk_*	–0.1211	–0.2276(15)	–0.2142	ϕ*_jk_*	0.0132	
ϕ*_k_*	0.4032		0.3582	ϕ*_k_*	–0.0453	

a Assymetrically reduced Hamiltonian.

b Symetrically reduced Hamiltonian.

The equilibrium parameters *B*_e_ were obtained from the geometries optimized using the accurate highly correlated method. Δ*B*^vib^, the vibrational contribution, was determined from the VPT2 α_*r*_^*i*^ vibration–rotation interaction parameters and the MP2/AVTZ cubic force field.

The parameters of the most stable CGcg structure shown in Table 3 were obtained to be *A*_0_ = 17233.99 MHz, *B*_0_ = 5572.58 MHz, and *C*_0_ = 4815.55 MHz, in very good agreement with the available laboratory data.^11^ Differences between theoretical and experimental data are only Δ*A*_0_ = −3.96 MHz, Δ*B*_0_ = 4.76 MHz and Δ*C*_0_ = 2.51 MHz. In the case of Tgc, for which experimental data exist, differences reach Δ*A*_0_ = 57.53 MHz, Δ*B*_0_ = −0.12 MHz, and Δ*C*_0_ = 0.16 MHz, which represent a very good agreement for *B*_0_ and *C*_0_, and a noticeable divergence for *A*_0_. This divergence can be attributed to both theory and experiments, given the challenges encountered in the assignments. Perhaps, similar problems to those found in the assignments of the CGcg′ conformer spectrum^11^ attributed by the authors of ref (11) to the strong interactions between torsional modes, difficult also the analysis of the Tgc spectrum. It has to be considered that theoretically the 3 rotational constants are computed together diagonalizing an inertia tensor obtained from a unique fully optimized geometry.

Quartic and sextic centrifugal distortion constants given in Table 3 are parameters of the Watson reduced Hamiltonian. They were computed from the MP2/AVTZ anharmonic force field. For CGcg, they are compared with previous experimental data of Motiyenko et al.^11^ As far as we know, experimental parameters are not available for the CGcg′ form.

### 2.5 Far-Infrared Spectrum

Many organic molecules show various interacting LAMs that cannot be treated separately. An example is MeOCH_2_OH, whose spectrum measured in the millimeter-wave range^11^ discloses a relevant coupling among torsional modes. The effect is strong enough to hinder the assignments using reduced effective Hamiltonians, depending on one or two vibrational coordinates.

**Figure 5:**
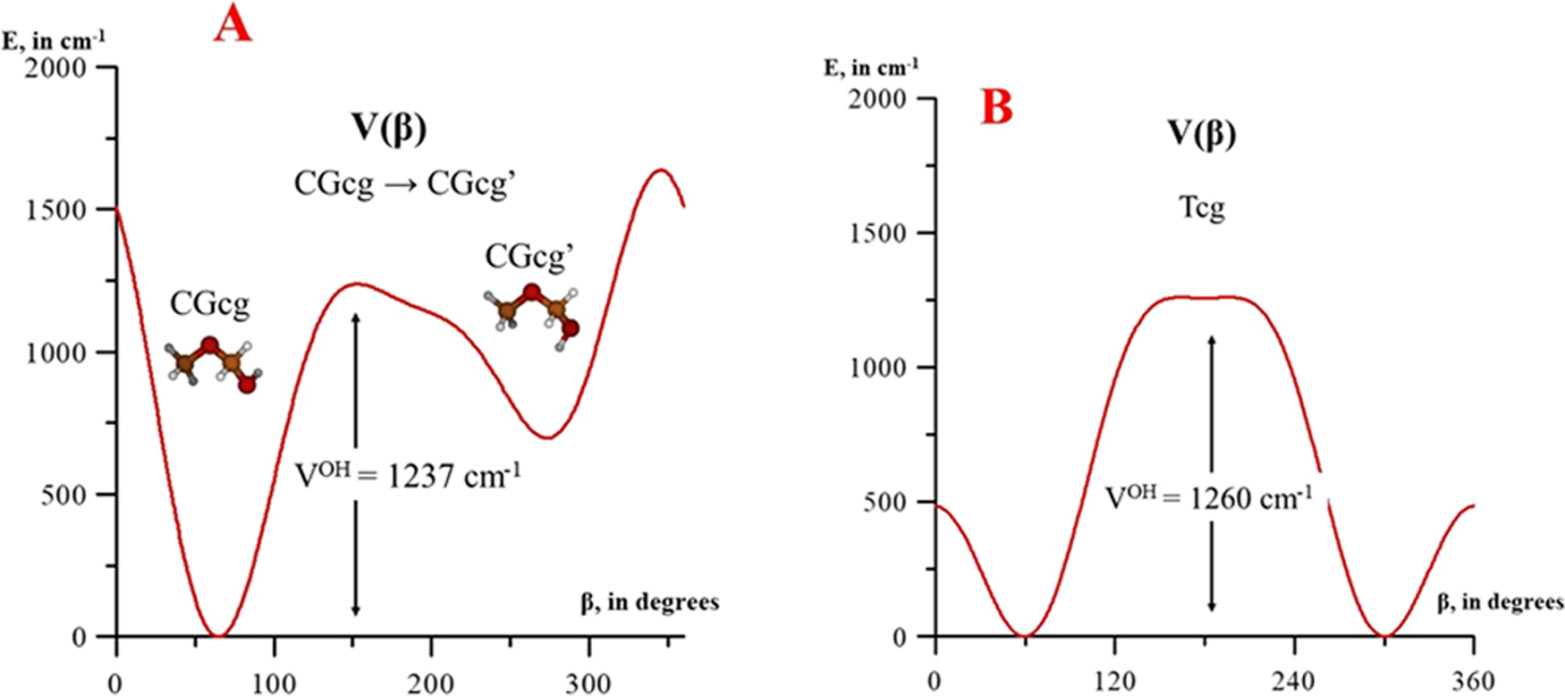
One-dimensional profiles depending on coordinate β (OH torsion). (A)^OH^ (CGcg → CGcg′); (B) V^OH^ (Tcg).

Figures 3–5 reveal that the barriers among the different conformers (<1500 cm^–1^) are of the same order of magnitude as the *V*_3_ methyl torsional barriers (600–900 cm^–1^). Vibrationally excited structures fall into the global minimum following almost barrier-less processes. The FIR transitions computed with VPT2 cannot be fully consistent because this theory assumes a single minimum, and the minimum interconversion can occur at very low temperatures. Within VPT2, the 3 conformers are treated as independent species.

However, on the basis of the VPT2 results (anharmonic constants and test of resonances), it is possible to assume that the three internal rotations are almost independent of the remaining vibrations. A variational procedure in three dimensions contemplating the conformer interconversion can be applied. Then, for *J* = 0, the torsional Hamiltonian can be defined as^32,33^
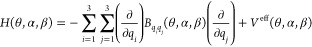
3where *q_i_*,*q_j_* = θ, α, and β; *B_q_i_q_j__* and *V*^eff^(θ, α, β) represent, respectively, the kinetic energy parameters and the effective potential defined as the sum of three contributions
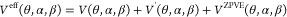
4Here *V*(θ, α, β) is the ab initio three-dimensional potential energy surface; *V*′(θ, α, β) represents the Podolsky pseudopotential; and *V*^ZPVE^(θ, α, β) is the harmonic zero-point vibrational energy correction.^30−32^

The ab initio three-dimensional potential energy surface, *V*(θ, α, β), was constructed using the CCSD(T)-F12/CVTZ-F12 energies of 157 geometries defined for selected values of *n* = 3 dihedral angles: H_4_C_2_O_1_C_3_ (0, ±90, 180°), O_7_C_3_O_1_C_2_ (0, 30, 60, 90, 120, 150, 180°), H_10_O_7_C_3_O_1_ (0, ±30, ±60, ±90, ±120, ±150, 180°). In all of the selected geometries, 3*N*-6-*n* internal coordinates (*N* = number of atoms; *n* = number of internal rotations) were optimized at the MP2/AVTZ level of theory. Details about the computation of the Podolsky pseudopotential *V*′(θ, α, β) and the kinetic energy parameters from the 157 optimized geometries can be found in refs (32) and (33). The zero-point vibrational energy correction *V*^ZPVE^(θ, α, β) was determined from the MP2/AVTZ *E*^ZPVE^ energies computed in all of the geometries with the harmonic approximation neglecting the contribution of the internal rotation modes.
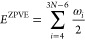
5

Although the pseudopotential is negligible, the *V*^ZPVE^(θ, α, β) contribution to the energy levels is significant.^36^ The 157 “effective” energies (*E*^eff^ = *E*^ab initio^ + *V*′+ *E*^ZPVE^) were fitted (σ = 0.1438; *R*^2^ = 0.9999) to a triple Fourier series transforming as the totally symmetric representation of the G_12_ Molecular Symmetry Group (MSG). The following equation was selected to analytically represent the effective potential
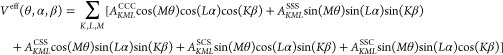
6

**Figure 6:**
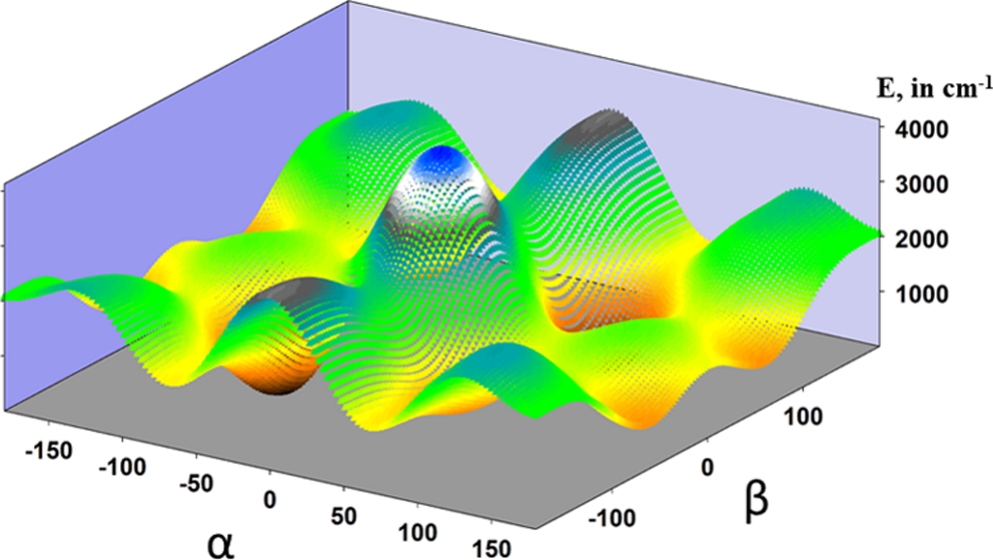
Two-dimensional potential energy surfaces depending on the coordinates α and β (θ = 180°).

The expansion coefficients of the effective potential are provided in Table S2. The comparison between independent and interaction terms indicates the strength of these interactions. Interaction terms between CH_3_ and the OH torsions are relatively small (i.e., 2.454 cos 3θ cos β), whereas those between the O–CH_3_ and the OH torsions are significant (i.e., 486.633 cos α cos β). Figure 6 represents a two-dimensional potential energy surface constructed by fixing the θ coordinate at 180°. The anisotropy of the surface describes the dependence between the O–CH_3_ and the OH torsions.

Analytical expressions containing 132 terms and formally identical to eq 6 were employed for the fitting of the kinetic energy parameters, *B*_*q_i_q_j_*_, computed in all of the geometries.^32,33^ The expansion coefficients are provided in the Supporting Information (see Table S3). The most contributed ones are the *A*_000_^CCC^ coefficients determined to be *A*_000_(*B*_θθ_) = 6.4272 cm^–1^, *A*_000_(*B*_αα_) = 2.4942 cm^–1^, *A*_000_(*B*_ββ_) = 22.2590 cm^–1^, *A*_000_(*B*_θα_) = −1.0907 cm^–1^, *A*_000_(*B*_θβ_) = −0.0180 cm^–1^, and *A*_000_(*B*_αβ_) = −0.7779 cm^–1^.

The final levels are obtained variationally using symmetry-adapted Fourier series as trial functions. Table 4 shows the energy levels localized in the CGcg minimum up to 700 cm^–1^ where they are compared to the VPT2 results. Low-lying energies assigned to the CGcg′ and Tcg minima are shown. The levels (000) for which *E*_000_ are their absolute energies were selected as origin of energies. The levels are classified by symmetry species of the G_12_ Molecular Symmetry Group (MSG). and three quanta. The latter are assigned considering the properties of the 3D-wave functions.^18,19^ The convergence requires diagonalizing matrices of at least 11,138 × 11,138 (*A*_1_), 11,137 × 11,137 (*A*_2_), and 20,250 × 20,250 (*E*). As a contracted basis set (21 symmetric and antisymmetric solutions of a 1D Hamiltonian depending on β) has been employed to describe the OH torsional wave function,^19^ the dimensionality has been reduced to 6962 × 6962 (*A*_1_), 6961 × 6961 (*A*_2_), and 12,348 × 12,348 (*E*), and the OH torsional excited levels have been unambiguously assigned.

**Table 4:** Low-Lying Vibrational Energy Levels of CH_3_OCH_2_OH (in cm^–1^) Referred to *E*_000_

CGcg-CH_3_OCH_2_OH							
υ_24_ υ_23_ υ_22_	variational		VPT2^a^	υ_24_ υ_23_ υ_22_	variational		VPT2^a^
0 0 0	*A*_1_, *A*_2_	0.0		3 1 0	*A*_1_, *A*_2_	537.491	628
	*E*	0.003			*E*	540.860	
1 0 0	*A*_1_, *A*_2_	132.133	135	0 3 0	*A*_1_, *A*_2_	543.078	571
	*E*	132.082			*E*	544.045	
0 1 0	*A*_1_, *A*_2_	186.507	194	0 1 1	*A*_1_, *A*_2_	555.948	515
	*E*	186.467			*E*	556.011	
2 0 0	*A*_1_, *A*_2_	261.433	270	υ_23_ υ_21_			580
	*E*	261.001					
1 1 0	*A*_1_, *A*_2_	309.095	323	2 2 0	*A*_1_, *A*_2_	590.203	628
	*E*	309.823			*E*	578.905	
0 2 0	*A*_1_, *A*_2_	366.299	385	5 0 0	*A*_1_, *A*_2_	620.067	678
	*E*	366.376			*E*	620.067	
						603.037	
0 0 1	*A*_1_, *A*_2_	371.921	337	2 0 1	*A*_1_, *A*_2_	628.488	610
	*E*	371.950	(260)		*E*	627.892	
υ_21_			384 (461)	2υ_24_ υ_21_			646
3 0 0	*A*_1_, *A*_2_	389.030	406	1 3 0	*A*_1_, *A*_2_	647.573	580
	*E*	384.600			*E*	642.906	
2 1 0	*A*_1_, *A*_2_	431.590	451	1 1 1	*A*_1_, *A*_2_	672.344	
	*E*	426.144			*E*	675.022	
4 0 0	*A*_1_, *A*_2_	479.313	542	0 4 0	*A*_1_, *A*_2_	680.844	753
	*E*	479.424			*E*	654.443	
1 2 0	*A*_1_, *A*_2_	482.135	506	6 0 0	*A*_1_, *A*_2_	688.928	
	*E*	506.736			*E*	675.022	
υ_24_ υ_21_			515	0 0 2	*A*_1_, *A*_2_	693.465	664
υ_20_			572		*E*	693.351	
1 0 1	*A*_1_, *A*_2_	500.617	473	*E*_000_	360.939		
	*E*	498.671					

CGcg′-CH_3_OCH_2_OH				Tcg-CH_3_OCH_2_OH			
υ_24_ υ_23_ υ_22_	variational		VPT2^a^	υ_24_ υ_23_ υ_22_	variational		VPT2^a^
0 0 0	*A*_1_, *A*_2_	0.0		0 0 0	*A*_1_	0.0	
					*A*_2_	2.477	
1 0 0	*A*_1_, *A*_2_	192.140	187	1 0 0	*A*_1_	203.565	216
					*A*_2_	203.505	
0 1 0	*A*_1_, *A*_2_	138.378	135	0 1 0	*A*_1_	106.397	109
					*A*_2_	106.347	
0 0 1	*A*_1_, *A*_2_	244.722	224	0 2 0	*A*_1_	202.496	212
					*A*_2_	202.085	
*E*_000_	988.817			*E*_000_	1135.350		

a *ν* (*ν* + Δ*ν*); Δ*ν* = predicted displacements due to Fermi and Darling–Dennison Resonances.

Due to the methyl torsion, the levels split into 3 components, one nondegenerate *A_i_* (*i* = 1,2) and two 2-fold degenerate *E*. In addition, since the most stable conformers show gauche structures and correspond to a double minimum at (θ, α, β) and (θ, −α, −β), each one of the methyl components splits into two subcomponents. In consequence, each level splits into 6 sublevels, two nongenerate *A*_1_, *A*_2_, and two 2-fold-degenerate *E*.

Symmetry constraints help in the classification and serve to reduce dimensionality. The levels can be assigned to the conformers, if probability integrals are computed from the three-dimensional wave functions^18,19^
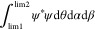
7

The main group of low-energy levels (below 700 cm^–1^) was assigned to the favor CGcg conformer. The *A*_1_ components of the ground vibrational state of the secondary conformers CGcg′ and Tcg were found at 627.879 and 744.411 cm^–1^ over the global ground vibrational state, respectively. Over 950 cm^–1^, it is difficult to distinguish the CGcg-levels, CGcg′-levels, and Tgc-levels because the wave functions are delocalized.

The *A*/*E* splitting of the ground vibrational state has been computed to be 0.003 cm^–1^, as was expected given the methyl torsional barrier (∼600 cm^–1^). The fundamental levels (100), (010), and (001) were found to lie at 132.133 and 132.082 cm^–1^ (methyl torsion), 186.507 and 186.467 cm^–1^ (O–CH_3_ torsion), and 371.921 and 371.950 cm^–1^ (OH torsion), respectively. The VPT2 corresponding energies were obtained to be 135, 194, and 337 cm^–1^. The latter is displaced to low frequencies (260 cm^–1^) if Darling–Dennison resonances are considered.

It is remarkable that for the CH_3_ torsion, the variational and VPT2 energies converge as a result of the harmonic character of this vibration. For example, the overtone 2*v*_24_ = 261.433 cm^–1^ is almost twice the fundamental ν_24_ = 132.133 cm^–1^ (2*v*_24_/*v*_24_ = 1.98). However, for the other two modes, 2*v*_23_/*v*_23_ = 1.96 and 2*v*_22_/*v*_22_ = 1.86. Subsequently, the energy (002) has been found to be 693.465 cm^–1^ (*A*_1_)/693.351 cm^–1^ (*E*).

A total of 24 × 6 = 144 torsional energies were found below 700 cm^–1^. It has to be highlighted that the gap between *A*_1_ and *A*_2_ levels is lower than 0.001 cm^–1^. In Table 4, the low-lying energy levels localized in the secondary minima are given. A small *A*_1_/*A*_2_ gap is obtained for Tcg- CH_3_OCH_2_OH.

Finally, the computed energies can be employed to determine the vibrational contributions to the partition functions applied in radiative transfer models for the interpretation of astrophysical observations.^25^ At low temperatures, different values of the vibrational contribution, *Q*_vib_, can be obtained using a theory developed for semirigid species (i.e., VPT2 which has been developed for species showing a single minimum in the potential energy surface) or theory for nonrigid species (i.e., the variational procedure which consider the minimum interconversion and the level splittings). Using our ab initio results, the vibrational partition functions were computed from the VPT2 torsional energies (*Q*_vib_) and from the variational torsional energies (*Q*_vib_^T^). In both cases, for the remaining vibrational modes, VPT2 anharmonic energies were employed (Table 5).

**Table 5:** Vibrational Partition Functions Computed from the VPT2 Torsional Energies (*Q*_vib_) and Computed Using the Variational Procedure (*Q*_vib_^T^)

*T* (K)	75	150	225	300	500	1000
*Q*_vib_	1.42	3.95	9.08	16.61	48.33	196.61
*Q*_vib_^T^	1.02	1.87	4.89	10.60	40.93	203.38

The ratio between *Q*_vib_ and *Q*_vib_^T^ has been estimated to be ∼1.4 below 100 K, ∼1.9 in the 100–500 K range, and ∼1.0 over 500 K.

## 3 Conclusions

In this paper, the structural and spectroscopic properties of MeOCH_2_OH are investigated using CCSD(T)-F12/CVTZ theory. Vibrational contributions to the rotational-torsional properties are determined at the MP2/AVTZ level of theory. The molecule presents three asymmetric conformers denoted by CGcg, CGcg′, and Tcg. CGcg represents the global minimum, although the relative energy of secondary conformers CGcg′ and Tcg are very low 641.5 and 792.7 cm^–1^. The computed ground vibrational state rotational constants are consistent with previous experimental data,^11^ with the exception of the *A*_0_ parameters of Tgc.

The three conformers are separated by relatively low barriers (ca. 1200–1500 cm^–1^) and interconvert through three large-amplitude motions which are the torsions of the CH_3_, OCH_3_, and OH groups. The methyl torsional barriers *V*_3_ of the conformers were estimated to be 595.7 cm^–1^ (CGcg), 829.0 cm^–1^ (CGcg′), and 807.5 cm^–1^ (Tgc), respectively. It can be concluded that the barriers that restrict the minimum interconversion are on the same order of magnitude (or lower) than the *V*_3_ barriers.

As was first inferred from the rotational spectrum assignments,^11^ methoxymethanol is a good example of organic systems showing various interacting LAMs that cannot be treated separately. The structural properties design a very flexible system and make necessary the study of the spectroscopy with models that consider the conformer interconversion. It can be concluded that the vibrationally excited structures produce the global minimum following almost barrier-less processes.

Each energy level computed variationally splits into six components, *A*_1_, *A*_2_, *E*, and *E*. The *A*/*E* splitting of the global ground vibrational state has been computed to be 0.003 cm^–1^, as was expected given the methyl torsional barrier (∼600 cm^–1^) of the CGcg conformer. For CGcg′ and Tcg, the splitting is very small. It must be emphasized that the gap between the *A*_1_ and *A*_2_ levels is lower than 0.001 cm^–1^ for energies lower than 700 cm^–1^.

At very low temperatures, the vibrational partition function *Q*_vib_ obtained from the variational torsional energy levels computed variationally is found to be lower than 10% than if it is computed using VPT2. This must be considered when radiative transfer models are applied for the interpretation of astrophysical observations.

## Supporting Information Available

CCSD(T)-F12/CVTZ-F12 and MP2/AVTZ minimum energy structures (Table S1); expansion coefficients of the 3D-potential energy surface (Table S2); and expansion coefficients of the kinetic energy parameters (Table S3) (PDF)
