# Supplementary material for: The Far-Infrared Spectrum of Methoxymethanol (CH3–O–CH2OH): A Theoretical Study
Source: ACS Earth Space Chem. 2024 May 29;8(6):1236–45. doi: 10.1021/acsearthspacechem.4c00053 (PMC11194847; doi:10.1021/acsearthspacechem.4c00053)
Supplement: Supplementary file 1 — sp4c00053_si_001.pdf [file sp4c00053_si_001.pdf]

Supporting Information for:

The Far Infrared Spectrum of Methoxy-methanol (CH3-O-CH2OH). A Theoretical Study

Dorsaf Missaoui[a,b], Sinda Brahem[a,b], Faouzi Najar[a], Ounaies Yazidi[a], María Luisa Senent[b*]

[a]Laboratoire de Spectroscopie Atomique Moléculaire et Applications, Faculté des Sciences de Tunis, Université de Tunis El Manar, 2092 Tunisia.
[b] Departamento de Química y Física Teóricas, Instituto de Estructura de la Materia, IEM-CSIC, Serrano 121, Madrid 28006; Unidad Asociada GIFMAN, CSIC-UHU, Spain; E_mail: ml.senent@csic.es

Table S1

CCSD(T)-F12/CVTZ-F12 and MP2/AVTZ minimum energy structures

Conformer 1
CCSD(T)-F12/CVTZ-F12 ENERGY=-230.24898350 a.u.

O	-0.5507594571	0.3178650742	-0.6447630626
C	0.5191808364	-0.1210967357	-1.4629633596
C	-0.5583936523	-0.3013981468	0.6031756676
H	0.4619806281	-1.2016832680	-1.6215855575
H	0.4182343282	0.3878598683	-2.4163923914
H	1.4839543013	0.1216319439	-1.0181330815
O	0.5287470778	0.0685919263	1.4095199887
H	-1.5077301673	-0.0266536527	1.0616322480
H	-0.4845440483	-1.3865624254	0.5008429966
H	0.4447904691	1.0056455450	1.5999298161

Conformer 2
CCSD(T)-F12/CVTZ-F12 ENERGY=-230.24581418 a.u.

O	-0.5666793942	0.2886597508	-0.6598016012
C	0.5307158384	-0.1065099261	-1.4527694603
C	-0.5579506389	-0.2957482668	0.6054979576
H	0.6050035461	-1.1986354894	-1.5008336643
H	0.3624838652	0.2817195283	-2.4524120549
H	1.4691473161	0.3012632404	-1.0706991023
O	0.4795454196	0.1620048711	1.4326464228
H	-1.4885046062	0.0003220360	1.0782747278
H	-0.5077385745	-1.3873788209	0.5073391817
H	1.2672582166	-0.3573916869	1.2670951055

Conformer3 3
CCSD(T)-F12/CVTZ-F12 ENERGY=-230.24485831 a.u.

O	0.0468892652	-0.4786558175	0.5130138323
C	-0.0244366014	0.1030841389	1.7950357108
C	-0.0077860508	0.4995194806	-0.4928455024
H	0.0050162375	-0.7055603537	2.5183055791
H	0.8212102280	0.7740834279	1.9734450924
H	-0.9530369329	0.6679332332	1.9252114283

O	0.0340084311	-0.1170411616	-1.7317620425
H	-0.9221032648	1.0957052659	-0.3615152437
H	0.8637325814	1.1573430639	-0.4397090659
H	-0.7149600545	-0.7146448491	-1.7875004736

Conformer 1
MP2/AVTZ

O						
C	1	1.422545				
C	1	1.398644	2	112.139		
H	2	1.093653	1	110.404	3	59.098
H	2	1.085620	1	106.798	4	118.891
H	2	1.089730	1	111.283	4	-121.335
O	3	1.409197	1	113.258	2	67.542
H	3	1.089486	1	105.524	7	122.317
H	3	1.092575	1	110.897	7	-117.792
H	7	0.963859	3	107.293	1	64.691

Conformer 2
MP2/AVTZ

O						
C	1	1.416193				
C	1	1.399135	2	112.440		
H	2	1.095660	1	110.809	3	52.960
H	2	1.085699	1	106.104	4	118.964
H	2	1.092017	1	111.493	4	-122.043
O	3	1.409602	1	113.831	2	68.639
H	3	1.085138	1	105.860	7	116.724
H	3	1.097146	1	109.615	7	-123.815
H	7	0.961743	3	108.907	1	-86.596

Conformer 3
MP2/AVTZ

O						
C	1	1.416064				
C	1	1.409952	2	110.711		
H	2	1.085487	1	107.117	3	179.437
H	2	1.094527	1	111.159	4	119.222
H	2	1.094527	1	111.281	4	-119.380
O	3	1.389973	1	109.260	2	181.228
H	3	1.099148	1	108.875	7	122.587
H	3	1.093250	1	110.600	7	-116.960
H	7	0.963733	3	107.470	1	59.433

Table S2

Expansion coefficients of the 3D-Potential Energy Surface (in cm^{-1})
(positive values of the M, L and K index denote cosine functions; negative values denote sine functions)

A_{MLK}	M	L	K	A_{MLK}	M	L	K
2224.458	0	0	0	335.548	3	0	0
-7.161	6	0	0	256.792	0	1	0
592.969	0	2	0	544.480	0	3	0
1.788	0	4	0	-221.217	0	0	1
371.891	0	0	2	196.819	0	0	3
-2.562	0	0	4	3.590	3	1	0
63.729	3	2	0	-19.221	3	3	0
-2.710	3	4	0	0.520	6	1	0
-5.195	6	2	0	-10.005	6	3	0
-2.237	6	4	0	-33.074	-3	-1	0
-109.279	-3	-2	0	-23.162	-3	-3	0
2.454	3	0	1	0.529	3	0	2
-1.246	3	0	3	0.402	3	0	4
0.891	6	0	1	0.335	6	0	2
0.312	6	0	3	-0.305	6	0	4
2.545	-3	0	-1	1.554	-3	0	-2
0.370	-3	0	-3	486.633	0	1	1
203.788	0	1	2	41.011	0	1	3
10.920	0	1	4	89.886	0	2	1
107.154	0	2	2	23.663	0	2	3
7.022	0	2	4	-23.113	0	3	1
-0.486	0	3	2	-1.325	0	3	3
-7.193	0	3	4	-6.452	0	4	1
-4.544	0	4	2	1.376	0	4	3
-1.942	0	4	4	-247.313	0	-1	-1
-164.783	0	-1	-2	-51.670	0	-1	-3
-130.433	0	-2	-1	-202.892	0	-2	-2
-18.121	0	-2	-3	-80.042	0	-3	-1
17.639	0	-3	-2	-3.630	0	-3	-3
21.979	3	1	1	-1.393	3	1	2
0.183	3	1	3	-1.247	3	1	4
20.241	3	2	1	21.191	3	2	2
3.156	3	2	3	1.319	3	2	4
31.630	3	3	1	36.642	3	3	2
14.169	3	3	3	1.003	3	3	4
9.223	3	4	1	13.619	3	4	2
8.350	3	4	3	1.662	3	4	4
-1.087	6	1	1	1.483	6	1	2
-0.611	6	1	3	0.343	6	1	4
-0.187	6	2	1	-0.148	6	2	2
1.132	6	2	3	0.085	6	2	4
-1.663	6	3	1	2.206	6	3	2
-0.215	6	3	3	1.159	6	3	4
0.324	6	4	1	1.208	6	4	2
0.792	6	4	3	0.120	6	4	4
-21.677	3	-1	-1	-4.767	3	-1	-2
-2.542	3	-1	-3	-37.990	3	-2	-1
-29.442	3	-2	-2	0.322	3	-2	-3
-24.393	3	-3	-1	-23.537	3	-3	-2
-0.627	3	-3	-3	1.182	6	-1	-1
1.486	6	-1	-2	0.421	6	-1	-3

1.315	6	-2	-1	2.130	6	-2	-2
0.493	6	-2	-3	1.701	6	-3	-1
1.760	6	-3	-2	0.526	6	-3	-3
-12.008	-3	-1	1	-1.081	-3	-1	2
1.666	-3	-1	3	0.583	-3	-1	4
-33.215	-3	-2	1	-19.779	-3	-2	2
1.318	-3	-2	3	0.849	-3	-2	4
-29.276	-3	-3	1	-27.206	-3	-3	2
1.069	-3	-3	3	0.252	-3	-3	4
-5.244	-3	1	-1	11.688	-3	1	-2
1.946	-3	1	-3	-28.141	-3	2	-1
-30.388	-3	2	-2	-8.247	-3	2	-3
-35.234	-3	3	-1	-36.164	-3	3	-2
-16.827	-3	3	-3	-13.896	-3	4	-1
-15.350	-3	4	-2	-11.302	-3	4	-3

Table S3

Expansion coefficients of the kinetic energy parameters (cm^{-1})

(positive values of the M, L and K index denote cosine functions; negative values denote sine functions)

$B_{\theta\theta}$	$B_{\alpha\alpha}$	$B_{\beta\beta}$	$B_{\theta\alpha}$	$B_{\theta\beta}$	$B_{\alpha\beta}$	**M**	**L**	**K**
6.4272	2.4942	22.2590	-1.0907	-0.0180	-0.7779	0	0	0
0.0563	0.0547	-0.0033	-0.0576	0.0005	-0.0023	3	0	0
0.0006	0.0004	0.0004	-0.0024	-0.0001	-0.0004	6	0	0
0.0712	0.8000	0.1898	-0.7214	1.0897	-0.8704	0	1	0
0.4295	0.4830	0.3061	-0.3644	0.0738	-0.2719	0	2	0
0.1337	0.2540	0.0145	-0.2134	0.1377	-0.1325	0	3	0
0.0423	0.0779	0.0309	-0.0468	0.0280	-0.0554	0	4	0
0.0146	0.0819	0.5297	-0.0189	-0.0012	-1.0490	0	0	1
0.0089	0.0220	0.1719	-0.0111	0.0040	0.0099	0	0	2
0.0008	0.0013	0.0938	-0.0020	0.0017	-0.0055	0	0	3
-0.0002	0.0017	0.0099	-0.0001	0.0008	-0.0038	0	0	4
0.0035	0.0452	0.0062	-0.0147	0.0155	-0.0319	3	1	0
0.0160	0.0338	0.0018	-0.0234	0.0062	-0.0111	3	2	0
0.0282	0.0456	0.0004	-0.0379	0.0034	-0.0114	3	3	0
0.0003	0.0130	-0.0014	-0.0046	0.0057	-0.0102	3	4	0
0.0006	0.0005	0.0001	-0.0006	-0.0006	0.0003	6	1	0
-0.0002	-0.0009	0.0001	0.0001	-0.0002	0.0003	6	2	0
-0.0021	-0.0027	0.0004	0.0022	0.0000	0.0008	6	3	0
-0.0004	-0.0015	-0.0001	0.0008	-0.0002	0.0008	6	4	0
-0.0103	-0.0178	0.0052	0.0141	-0.0012	0.0026	-3	-1	0
-0.0147	-0.0314	-0.0109	0.0166	-0.0060	0.0175	-3	-2	0
-0.0321	-0.0432	-0.0054	0.0388	-0.0020	0.0091	-3	-3	0
0.0039	0.0037	0.0026	-0.0042	0.0011	-0.0004	3	0	1
0.0011	0.0007	-0.0048	-0.0012	0.0010	-0.0002	3	0	2
0.0004	0.0005	-0.0025	-0.0006	0.0003	0.0005	3	0	3
-0.0001	-0.0000	-0.0014	0.0001	0.0001	0.0000	3	0	4
0.0004	0.0006	0.0012	-0.0005	-0.0000	-0.0001	6	0	1
-0.0001	0.0002	0.0001	0.0000	0.0000	-0.0003	6	0	2
-0.0001	-0.0003	0.0004	0.0003	-0.0000	-0.0002	6	0	3
-0.0000	0.0001	0.0001	0.0000	0.0000	-0.0001	6	0	4
-0.0004	-0.0015	0.0010	0.0010	-0.0034	0.0035	-3	0	-1
-0.0002	-0.0007	-0.0009	0.0006	-0.0012	0.0008	-3	0	-2
-0.0003	-0.0009	0.0006	0.0006	-0.0005	0.0005	-3	0	-3
0.0269	0.1022	0.4388	-0.0519	-0.0177	-0.0216	0	1	1

0.0175	0.0098	0.2533	-0.0072	-0.0001	-0.0322	0	1	2
0.0026	0.0087	0.0855	-0.0034	0.0034	-0.0156	0	1	3
-0.0001	0.0019	0.0239	-0.0006	0.0014	-0.0048	0	1	4
0.0095	0.0464	0.1203	-0.0214	-0.0017	-0.0178	0	2	1
0.0327	0.0369	0.1682	-0.0330	0.0034	-0.0125	0	2	2
0.0047	0.0094	0.0542	-0.0062	0.0001	-0.0098	0	2	3
0.0004	0.0016	0.0205	-0.0005	-0.0005	-0.0023	0	2	4
-0.0015	0.0125	0.0115	-0.0029	-0.0045	0.0016	0	3	1
0.0080	0.0163	-0.0097	-0.0120	0.0085	-0.0069	0	3	2
0.0038	0.0076	-0.0088	-0.0053	0.0007	0.0001	0	3	3
0.0001	0.0003	-0.0086	-0.0001	-0.0008	0.0015	0	3	4
-0.0018	0.0026	-0.0117	-0.0003	-0.0022	0.0018	0	4	1
0.0051	0.0049	0.0054	-0.0049	0.0009	-0.0001	0	4	2
0.0021	0.0033	-0.0046	-0.0024	0.0002	-0.0005	0	4	3
-0.0001	-0.0002	-0.0044	0.0001	-0.0003	0.0007	0	4	4
-0.0177	-0.0160	0.0078	0.0190	0.7641	-0.6240	0	-1	-1
-0.0050	0.0372	-0.1192	-0.0257	0.0049	0.0039	0	-1	-2
-0.0018	-0.0050	-0.0613	0.0022	0.0062	0.0006	0	-1	-3
-0.0162	-0.0307	0.4240	0.0171	0.0411	-0.1516	0	-2	-1
-0.0009	0.0002	-0.1083	0.0014	0.0038	-0.0059	0	-2	-2
-0.0055	-0.0088	-0.0422	0.0063	0.0025	0.0042	0	-2	-3
-0.0147	-0.0215	0.0571	0.0174	0.0788	-0.0714	0	-3	-1
-0.0020	0.0005	-0.0034	-0.0009	0.0019	-0.0039	0	-3	-2
-0.0024	-0.0051	-0.0059	0.0029	0.0023	-0.0010	0	-3	-3
0.0127	0.0114	0.0101	-0.0117	0.0011	-0.0013	3	1	1
0.0041	0.0030	-0.0062	-0.0043	0.0023	-0.0010	3	1	2
0.0008	0.0011	-0.0047	-0.0012	0.0009	0.0002	3	1	3
0.0002	0.0003	-0.0012	-0.0003	0.0001	0.0001	3	1	4
0.0154	0.0135	0.0099	-0.0125	0.0007	-0.0025	3	2	1
0.0104	0.0092	0.0014	-0.0086	0.0024	-0.0036	3	2	2
0.0028	0.0028	0.0012	-0.0025	0.0009	-0.0011	3	2	3
0.0009	0.0007	0.0004	-0.0007	0.0001	-0.0000	3	2	4
0.0127	0.0133	0.0141	-0.0098	0.0012	-0.0044	3	3	1
0.0147	0.0134	0.0127	-0.0110	0.0015	-0.0046	3	3	2
0.0061	0.0065	0.0122	-0.0050	0.0008	-0.0025	3	3	3
0.0015	0.0012	0.0023	-0.0010	0.0002	-0.0006	3	3	4
0.0057	0.0068	0.0046	-0.0047	0.0003	-0.0022	3	4	1
0.0064	0.0070	0.0064	-0.0049	0.0005	-0.0021	3	4	2
0.0041	0.0046	0.0093	-0.0036	0.0004	-0.0013	3	4	3
0.0011	0.0009	0.0029	-0.0007	0.0001	-0.0004	3	4	4
0.0004	0.0009	0.0005	-0.0008	-0.0000	-0.0003	6	1	1
0.0004	0.0008	0.0004	-0.0004	0.0000	-0.0007	6	1	2
-0.0002	-0.0002	0.0006	0.0003	0.0000	-0.0004	6	1	3
0.0002	0.0003	0.0009	-0.0002	-0.0000	-0.0002	6	1	4
0.0006	0.0004	-0.0007	-0.0006	0.0001	-0.0002	6	2	1
0.0008	0.0011	0.0006	-0.0009	0.0001	-0.0003	6	2	2
0.0006	0.0009	0.0010	-0.0007	0.0000	-0.0001	6	2	3
0.0002	0.0003	0.0008	-0.0002	0.0000	-0.0001	6	2	4
0.0009	0.0002	-0.0015	-0.0007	0.0002	-0.0003	6	3	1
0.0014	0.0015	0.0022	-0.0016	0.0002	-0.0002	6	3	2
0.0010	0.0010	0.0016	-0.0011	0.0000	0.0000	6	3	3
0.0002	0.0003	0.0007	-0.0003	0.0001	-0.0002	6	3	4
0.0006	0.0003	-0.0011	-0.0005	0.0001	-0.0001	6	4	1
0.0013	0.0009	0.0017	-0.0011	0.0001	-0.0002	6	4	2
0.0002	0.0001	0.0007	-0.0002	0.0001	-0.0001	6	4	3
0.0003	0.0003	0.0008	-0.0003	0.0000	-0.0001	6	4	4
-0.0080	-0.0091	-0.0064	0.0082	0.0115	-0.0154	3	-1	-1
-0.0030	-0.0014	-0.0066	0.0028	-0.0014	0.0003	3	-1	-2

-0.0004	0.0000	-0.0019	0.0003	-0.0004	0.0005	3	-1	-3
-0.0134	-0.0130	-0.0121	0.0118	-0.0050	0.0068	3	-2	-1
-0.0090	-0.0047	-0.0135	0.0059	-0.0008	0.0006	3	-2	-2
-0.0011	-0.0001	-0.0010	0.0006	-0.0008	0.0010	3	-2	-3
-0.0112	-0.0110	-0.0226	0.0095	0.0016	0.0009	3	-3	-1
-0.0063	-0.0027	-0.0120	0.0032	-0.0002	0.0014	3	-3	-2
-0.0010	0.0001	-0.0006	0.0005	-0.0002	0.0001	3	-3	-3
-0.0000	0.0001	-0.0003	0.0001	-0.0000	0.0005	6	-1	-1
0.0000	-0.0000	0.0001	-0.0000	0.0001	0.0003	6	-1	-2
-0.0002	-0.0003	0.0004	0.0003	0.0001	-0.0001	6	-1	-3
-0.0003	-0.0002	-0.0013	0.0006	0.0005	-0.0004	6	-2	-1
-0.0001	0.0000	-0.0001	0.0003	0.0001	0.0001	6	-2	-2
0.0001	-0.0001	0.0004	0.0001	-0.0000	-0.0000	6	-2	-3
-0.0003	-0.0006	-0.0011	0.0007	0.0002	-0.0002	6	-3	-1
-0.0001	-0.0003	0.0005	0.0005	-0.0001	0.0001	6	-3	-2
0.0001	-0.0000	0.0005	-0.0000	0.0000	0.0001	6	-3	-3
-0.0094	-0.0072	-0.0100	0.0078	-0.0005	0.0008	-3	-1	1
-0.0035	-0.0034	0.0040	0.0031	-0.0016	0.0022	-3	-1	2
-0.0003	-0.0001	0.0007	0.0004	-0.0007	0.0002	-3	-1	3
0.0001	0.0003	0.0009	0.0000	-0.0003	0.0001	-3	-1	4
-0.0144	-0.0114	-0.0316	0.0108	-0.0000	0.0034	-3	-2	1
-0.0083	-0.0053	-0.0033	0.0062	-0.0020	0.0029	-3	-2	2
-0.0010	-0.0001	0.0007	0.0008	-0.0009	0.0005	-3	-2	3
0.0001	0.0004	0.0003	-0.0001	-0.0005	0.0003	-3	-2	4
-0.0091	-0.0077	-0.0259	0.0062	-0.0001	0.0030	-3	-3	1
-0.0078	-0.0048	-0.0095	0.0051	-0.0007	0.0022	-3	-3	2
-0.0008	0.0001	-0.0008	0.0003	-0.0006	0.0007	-3	-3	3
-0.0001	0.0002	0.0008	0.0000	-0.0004	0.0004	-3	-3	4
-0.0066	-0.0068	0.0055	0.0071	-0.0034	0.0040	-3	1	-1
-0.0019	-0.0034	-0.0013	0.0030	-0.0028	0.0017	-3	1	-2
-0.0015	-0.0030	0.0033	0.0022	-0.0012	0.0015	-3	1	-3
-0.0118	-0.0133	-0.0172	0.0101	-0.0057	0.0095	-3	2	-1
-0.0094	-0.0076	-0.0118	0.0065	-0.0019	0.0035	-3	2	-2
-0.0037	-0.0053	-0.0056	0.0041	-0.0015	0.0024	-3	2	-3
-0.0173	-0.0200	-0.0220	0.0162	-0.0004	0.0037	-3	3	-1
-0.0130	-0.0124	-0.0203	0.0094	-0.0009	0.0043	-3	3	-2
-0.0060	-0.0080	-0.0157	0.0057	-0.0012	0.0029	-3	3	-3
-0.0051	-0.0091	-0.0136	0.0051	0.0028	-0.0015	-3	4	-1
-0.0062	-0.0064	-0.0125	0.0043	-0.0004	0.0026	-3	4	-2
-0.0041	-0.0049	-0.0108	0.0037	-0.0002	0.0013	-3	4	-3
